# The Impairment of ILK Related Angiogenesis Involved in Cardiac Maladaptation after Infarction

**DOI:** 10.1371/journal.pone.0024115

**Published:** 2011-09-16

**Authors:** Jun Xie, Wen Lu, Rong Gu, Qin Dai, Bin Zong, Lin Ling, Biao Xu

**Affiliations:** Department of Cardiology, Drum Tower Hospital, Nanjing University Medical School, Nanjing, China; Northwestern University, United States of America

## Abstract

**Background:**

Integrin linked kinase (ILK), as an important component of mechanical stretch sensor, can initiate cellular signaling response in the heart when cardiac preload increases. Previous work demonstrated increased ILK expression could induce angiogenesis to improved heart function after MI. However the patholo-physiological role of ILK in cardiac remodeling after MI is not clear.

**Method and Results:**

Hearts were induced to cardiac remodeling by infarction and studied in Sprague-Dawley rats. Until 4 weeks after infarction, ILK expression was increased in non-ischemic tissue in parallel with myocytes hypertrophy and compensatory cardiac function. 8 weeks later, when decompensation of heart function occurred, ILK level returned to baseline. Followed ILK alternation, vascular endothelial growth factor (VEGF) expression and phosphorylation of endothelial nitric oxide synthase (eNOS) was significantly decreased 8 weeks after MI. Histology study also showed significantly microvessel decreased and myocytes loss 8 weeks paralleled with ILK down-regualtion. While ILK expression was maintained by gene delivery, tissue angiogenesis and cardiac function was preserved during cardiac remodeling.

**Conclusion:**

Temporally up-regulation of ILK level in non-ischemic myocytes by increased external load is associated with beneficial angiogenesis to maintain infarction-induced cardiac hypertrophy. When ILK expression returns to normal, this cardiac adaptive response for infarction is weaken. Understanding the ILK related mechanism of cardiac maladaptation leads to a new strategy for treatment of heart failure after infarction.

## Introduction

Coronary artery disease, especially occlusion of cardiac vessels is known as the most predominant reason for heart failure. After infarction, impaired heart contractility induces myocardium compensatory hypertrophy to attenuate heart dysfunction [1], [2], [3]. However compensatory hypertrophy after myocardial infarction (MI) is temporary. In the end, cardiac dilation and myocardium thinning occurs and heart function is further deteriorated.

Angiogenesis is very important to preserve adapt cardiac hypertrophy and contractile function. Neo-vascular could support more oxygen and energy to hypertrophic myocardium in non-ischemic zone [4], [5]. When oxygen and energy support is insufficient for hypertrophical myocytes, apoptosis or/and necrosis of cell would occur, leading to myocardium thinning and cardiac contractile impairment. Therefore, relatively insufficient microvessel is a key pathological feature in the transition from adaptive cardiac hypertrophy to cardiac dysfunction.

Integrin linked kinase (ILK) is a serine/threonine protein kinase, as a downstream kinase of β- integrin. It is an important component of mechanical stretch sensor, which can transduce mechanical signal to intra-cellular biomechanical signals, and initiate a serial of cellular responses [6], [7]. When cardiac pre-load increased, ILK is thought to transduce cardiac pressure load into a cellular compensatory growth program via activation of the Rho family guanine triphosphatases, ras-related C3 botulinum toxin substrate 1 (Rac1) and Cell division control protein 42 (Cdc42) [8]. ILK is also observed to promote vascular development and angiogenesis. In ILK knockout mice and zebrafish, the vasculature formation is markedly abnormal [9]. Vascular endothelial growth factor (VEGF) was identified as downstream of ILK to induce tissue angiogenesis. Several study revealed ILK was involved in cancer cell VEGF expression and tumor angiogenesis [10].

In our pervious work, overexpression of ILK after myocardial infarction can significantly induced myocardium hypertrophy and tissue angiogenesis to improve heart function [11]. However the patholo-physiological role of ILK in cardiac remodeling after MI has not been clear. Whether impairment of ILK related signaling pathway is the key feature for transition from adaptive cardiac hypertrophy to cardiac dysfunction needs to be clarified. To this aim, we assessed time-dependent ILK expression in non-ischemic myocardium after MI. We observed ILK as well as its related pro-angiogenic signaling down-regulated during the transition from adaptive cardiac hypertrophy to cardiac dysfunction; while the sustained ILK expression could maintain responsible angiogenesis in myocardium and improve heart function.

## Methods

### Experimental design

Animal experiments were performed following the guidelines in the Guide for the Care and Use of Laboratory Animals published by the US National Institutes of Health (NIH publication No. 85-23, revised 1985) and was approved by the Ethics Review Board for Animal Studies of Nanjing Drum Tower Hospital (DTH ERBA 66.01/005A/2010). Adult, male Sprague-Dawley rats (n = 40) weighing 200 to 220 g were used in this study. Left anterior descending coronary artery (LAD) ligation was performed as described previously in detail [11]. Sham-treated animals underwent open chest surgery without coronary artery ligation. 6 MI rats and 6 sham rats were sacrificed at day 7, 28 and 56 after myocardial infarction (MI). Before sacrificed transthoracic echocardiography and cardiac catheterization were performed to evaluate heart function. To evaluate the therapeutic effect of sustained ILK expression in cardiac remodeling, 19 rats underwent MI and were enrolled in study. 4 weeks after MI, the rats were thoracotomy again. Ad-ILK or Ad-null was injected into remote myocardium randomly and 12 rats were survived from the operation (6 rats per group). Two weeks after operation, we measured heart function by ECG, and executed rats to harvest myocardium for western blot analysis.

### Echocardiography and Hemodynamic Assessment

We monitored the cardiac function using transthoracic echocardiography and hemodynamic assessment at varying intervals (day 7, 28 and 56 post operation).

Transthoracic echocardiography was performed using a cardiovascular ultrasound system (Vivid 7, General Electric Co.) with a 10-MHz linear-array transducer as described previously [11]. Briefly, after anesthesia, rats are placed on a warm blanket. We obtained the long-axis and short-axis 2-dimensional views as well as the M-mode tracings. The measurements of left ventricular end-systolic dimension (LVESD) and end-diastolic dimension (LVEDD) as well as the interventricular septal thickness in diastole (IVSd) and LV posterior wall thickness in diastole (LVPWd) were all obtained in the M-mode tracings at the papillary muscle level by an observer blinded to the surgery, which were repeated for at least three consecutive pulsation cycles. The averaged data were used for analysis. Percent LV fractional shortening (%FS) was obtained as follows: FS = (LVEDD-LVESD)/LVEDD×100 (%).

After echocardiography, hemodynamic assessment was performed using a polyethylene catheter (PE 50, Becton-Dickinson) as described previously [12]. LV systolic pressure (LVSP), LV end-diastolic pressure (LVEDP), maximum positive dp/dt (+dp/dtmax) and heart rates (HR) were recorded continuously. All measurements were blinded to the observer.

### Histology test

After hemodynamic evaluation, hearts were arrested in diastole by intravenous administration of 2 M KCl, rapidly excised and washed in ice-cold saline. The hearts were incised into two parts in longitudinal axis. Half was embedded in paraffin wax. The other was homogenized for western blot. Paraffin-embedded sections (from basal, mid-region, and apical blocks, 5 slices/block) were stained with hematoxylin-eosin for morphologic examination. Von Willebrand factor (vWF) was used to detect microvessels, The paraffin-embedded sections (2-um slices) were incubated overnight with the rabbit-anti-rat vWF antibody (1∶400, Abcom, USA),then exposed to the secondary antibody (Dako, Northern Ireland). The images were analyzed by two investigators who were blinded with respect to the identification of the groups. The results were expressed as average numbers of capillaries per 10× field [11].

### Western blot procedure

Remote myocardium was subsequently homogenized with a Dounce tissue grinder in lysis buffer consisting of: 10 mM Tris/HCl (pH 7.2), 150 mM NaCl, 0.1%SDS, 1% (v/v) Triton X-100, 1% sodium deoxycholate, 5 mM EDTA, 1 mM Na3VO4, 50 mM NaF, 0.2 mM phenymethylsulfonyl fluoride and protease inhibitor cocktail (complete EDTA-free, Roche). Protein content was determined by BCA protein assay kit (Pierce). Proteins were separated by SDS-polyacrylamide gel electrophoresis (SDS-PAGE) and transferred to polyvinylidene fluoride membranes (Immobilon-P, Millipore). Western blots were probed with mouse anti-human ILK antibody (1∶3000, BD Transduction Laboratories), mouse anti-rat VEGF antibody (1∶1000, Santa Cruz), mouse anti-eNOS (1∶1000, BD Transduction Laboratories), mouse anti-p-eNOS (1∶100, BD Transduction Laboratories), rabbit anti-Akt (1∶2000, Cell Signaling Technology), rabbit anti-p-Akt (1∶100, Cell Signaling Technology) or mouse anti-GAPDH (1∶3000, Kangchen Bio-tech). Peroxidase-conjugated goat anti-mouse IgG or goat anti-rabbit IgG (1∶10,000, Santa Cruz Biotechnology) was used as secondary antibody, and bands were visualized by enhanced chemiluminescence (Amersham) or Chemiluminescence HRP substrate (Millipore) in conjunction with BioMax films (Kodak).

### Quantitative Real-Time PCR

Total RNA from remote myocardium was prepared by Trizol (Invitrogen) using protocols provided by the manufacturer. cDNA was produced using PrimeScript^tm^ RT reagent Kit (TaKaRa). Real-time PCR was performed as described previously [13]. Transcript levels of integrin linked kinase (ILK) was determined as the relative number of transcripts to those of glyceraldehydes-3-phosphate dehydrogenase and normalized to the mean value of control hearts. Primers for ILK and glyceraldehydes-3-phosphate dehydrogenase: ILK, CAACCCTCATCACACACTGG (forward), GCCATGTCCAAAGCAAACTT (reverse); ANF: CTTGCGGTGTGTCACACAGC (forward), GGGAGAGGTAAGGCCTCACT (reverse); GAPDH: AACGACCCCTTCATTGAC (forward), TCCACGACATACTCAGCAC (reverse).

### Data analysis

Descriptive statistics are expressed as group means+standard error. All data analysis was performed by ANOVA with Dunnett's post-test. All data were analysed using of SPSS 16.0 statistical software (SPSS, Inc, Chicago, IL, USA). Statistical significance was defined as p<0.05.

## Results

### Cardiac function was significantly deteriorated 8 weeks post-MI

In this study, we ligated left anterior descending coronary artery to achieve experimental rat MI model. After MI, we used echocardiograph and hemodynamic assessment to assess heart function. We observed cardiac function was maintained until 4 weeks, and significantly deteriorated 8 weeks post-MI (Table 1). In echocardiography (ECG) assessment, impaired cardiac function measured as FS were observed after MI. Left ventricular end systolic and diastolic diameters (LVEDD and LVESD) were increased significantly and consistently during cardiac remodeling after infarction. LV posterior wall in diastolic (LVPWd), which was non-ischemic myocardium, were moderately thicker compared with sham 4 weeks after MI, and markedly decreased 8 weeks after MI. Hemodynamic assessment showed mean artery pressure, dp/dt_max_ and LV end-diastolic pressure (LVEDP), but no HR, were markedly altered after MI. The global LV systolic function, as assessed by dp/dt_max_ and LVEDP was little changed until 4 weeks after MI, while it significantly deteriorated 8 weeks post MI. These results indicated that the shift from heart functional compensatory to decompensatory occurred during 4 to 8 weeks post-MI.

**Table 1:** Heart function after MI at different time points (n = 6).

	1 week		4 week		8 week	
	sham group	MI	sham group	MI	sham group	MI
**LVEDD(mm)**	5.58±0.32	6.53±0.4* #	5.32±0.28	7.86±0.28* #	5.54±0.63	9.12±0.36*
**LVESD(mm)**	2.87±0.21	4.79±0.31* #	2.95±0.12	5.75±0.22* #	2.96±0.51	6.88±0.38*
**IVSd(mm)**	1.19±0.08	1.22±0.04#	1.21±0.04	1.17±0.03#	1.26±0.02	1.04±0.09*
**LVPWd(mm)**	1.2±0.044	1.14±0.02#	1.23±0.05	1.22±0.03#	1.18±0.04	1.2±0.01*
**FS(%)**	48.8±1.45	26.61±0.29*	47.69±3.2	26.86±1.97*	43.91±5.25	24.62±2.3*
**HR(bpm)**	407.13±13.462	384.5±21.88	432.45±32.13	438.33±41.89	408.81±22.57	451.7±43.82
**MAP(mmHg)**	105.41±7.15	70.47±4.56*	103.67±6.35	81.75±8.6	113.15±5.98	71.15±5.27*
**dp/dtmax(mmHg/s)**	3341.7±158.07	1522.3±116.32*	3547±213.45	2009.8±152.62* #	3091.43±287.45	1195.4±267.38*
**LVEDP(mmHg)**	0.73±0.92	8.52±1.49* #	0.35±1.35	6.17±1.18* #	0.47±0.74	19.33±3.82*

# P<0.05 compared by group of 8 wks post MI.

*P<0.05 compared by sham group at each time point.

### ILK and angiogenesis related molecular expression was reduced at 8 weeks post-MI

**Figure 1:**
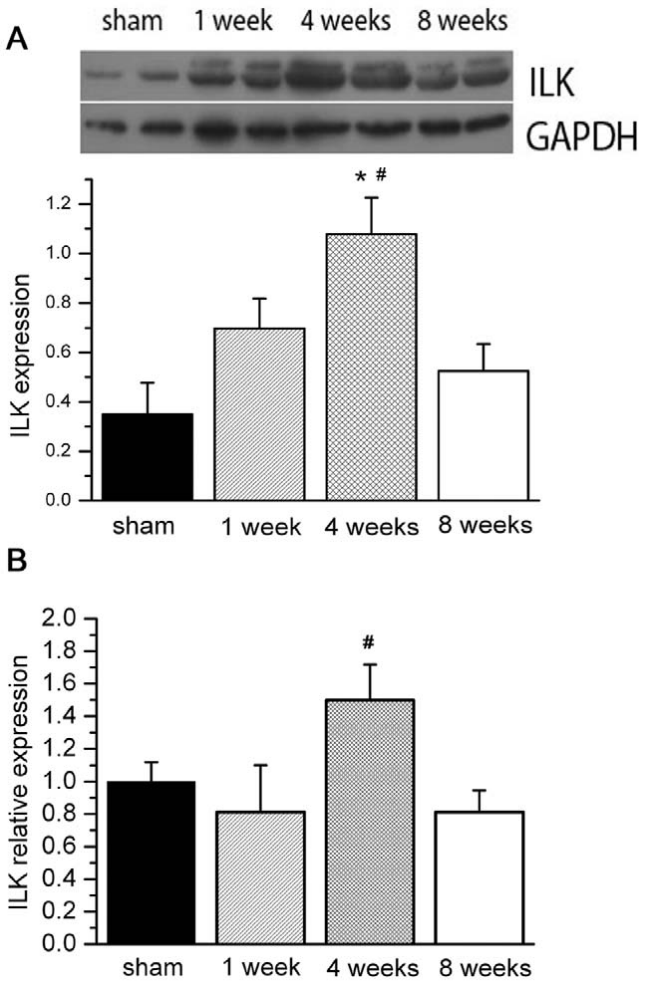
ILK expression in remote myocardium at different time points. A, in western blot, ILK protein level is significantly increased 4 weeks after MI, and return to normal 8 weeks after MI; B, in parallel with protein level, ILK mRNA expression is significantly increased 4 wks post MI (n = 6). # P<0.05 compared by group of 8 wks post MI. * P<0.05 compared by sham group.

We observed LV mechanical stretch, measured as LVEDP, was consistently increased during cardiac remodeling after MI. Since ILK was a key kinase in mechanical stretch sensor, we wanted to assess its change in myocardium during the cardiac remodeling process. Western blot and real time PCR were used to assess ILK expression. We observed that ILK expression in non-ischemic myocardium was increased and went to peak around 4 weeks post-MI, followed by a decline and eventually tapered off by 8 weeks (Fig. 1A). Consistent with protein alternation, ILK mRNA expression was also significantly deceased 8 weeks post MI (Fig. 1B). This alternation of ILK expression occurred during 4 weeks to 8 weeks after MI, paralleled with onset of cardiac function decompensatory.

**Figure 2:**
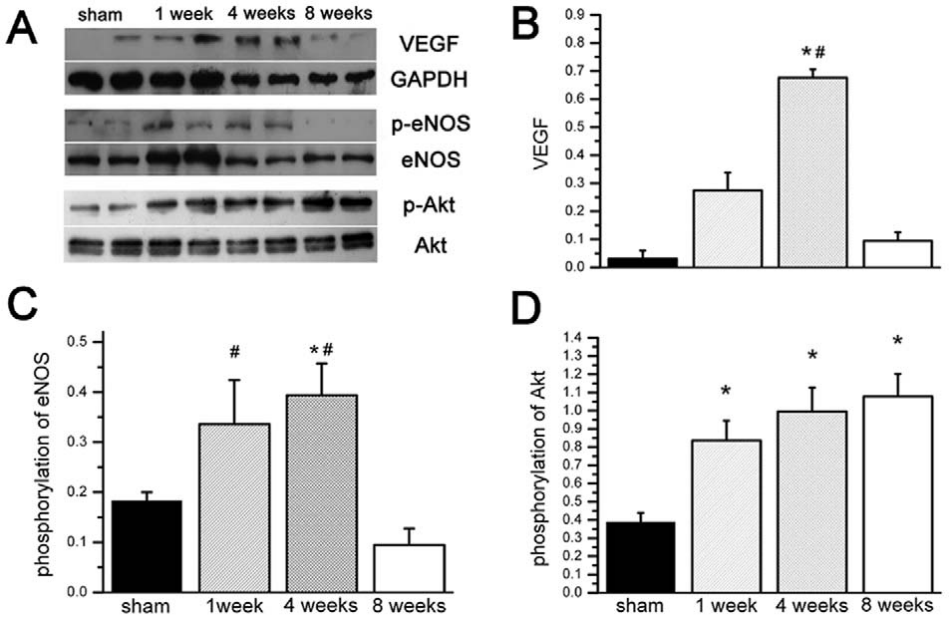
Alternation of angiogenesis signal in remote cardiac tissue at different time points. A, Representative western blot results of VEGF, p-eNOS and p-Akt; B, After MI, VEGF protein expression is increased until 4 wks, and decreased significantly 8 wks post MI; C, Phosphorylation of eNOS is also significantly increased after MI. At 8 wks post MI, p-eNOS level is decreased significantly; D, Phosphorylation of AKT level is significantly increased after MI, but not reduced at 8 weeks post MI (n = 6). # P<0.05 compared by group of 8 wks post MI. * P<0.05 compared by sham group.

Because ILK could induce VEGF expression to promote angiogenesis [10], we further measured VEGF, p-eNOS/eNOS and p-Akt/Akt level in myocardium. We observed VEGF and angiogenesis key kinase eNOS in the remote myocardium were changed followed ILK expression up and down. As most important endothelial cell mitogen [14], VEGF expression in the remote myocardium went to peak 4 weeks and returned to baseline 8 weeks post MI (Fig. 2B). Angiogenesis key kinase, eNOS was also significantly phosphorylated until 4 weeks post MI, but was dephosphorylated 8 weeks post MI (Fig. 2C). We further assessed ILK downstream kinase Akt activity. Phosphorylated Akt level was significantly increased after MI, but no significant alternation was observed among three time points after MI (Fig. 2D). All these results suggested angiogenesis was activated consistently for about 4 weeks after infarction. When ILK expression was down-regulated, angiogenesis related signaling pathway was also weakened.

### Reduced myocytes and microvessel were observed 8 weeks post MI

**Figure 3:**
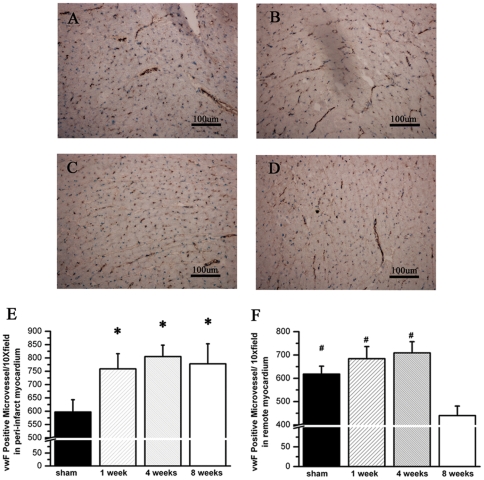
Microvessel density is significantly decreased in the remote myocardium eight weeks post MI. A to D, Representative tissue slide from remote myocardium with vWF staining in sham (A), one week (B), four weeks (C) and eight weeks group (D); E, Accumulated results show microvessel density is significantly increased in peri-infarct zone after MI. No temporal changes of microvessel density in peri-infarct myocardium is observed after MI. F, Accumulated results show microvessel density in remote myocardium is moderately increased after MI, and significantly decreased eight weeks post MI (n = 4). # P<0.05 compared by group of 8 wks post MI. * P<0.05 compared by sham group.

Angiogenesis pathway was down-regulated in non-ischemic myocardium 8 weeks post MI. Furthermore we assessed the microvessel density by histological analysis. von Willebrand factor (vwF) was selected as endothelial cell biomarker in immune-staining. In peri-infarct zone, microvascular density was significantly increased in the MI group compared with sham group. No difference of microvessel density was observed among three time points after MI (Fig. 3E). In remote zone, microvessel density was moderately increased after MI, but not reached significantly difference compared with sham. In rats executed 8 weeks after MI, the microvessel density was significantly decreased compared with those in rats executed other time spots after MI (Fig. 3F).

**Figure 4:**
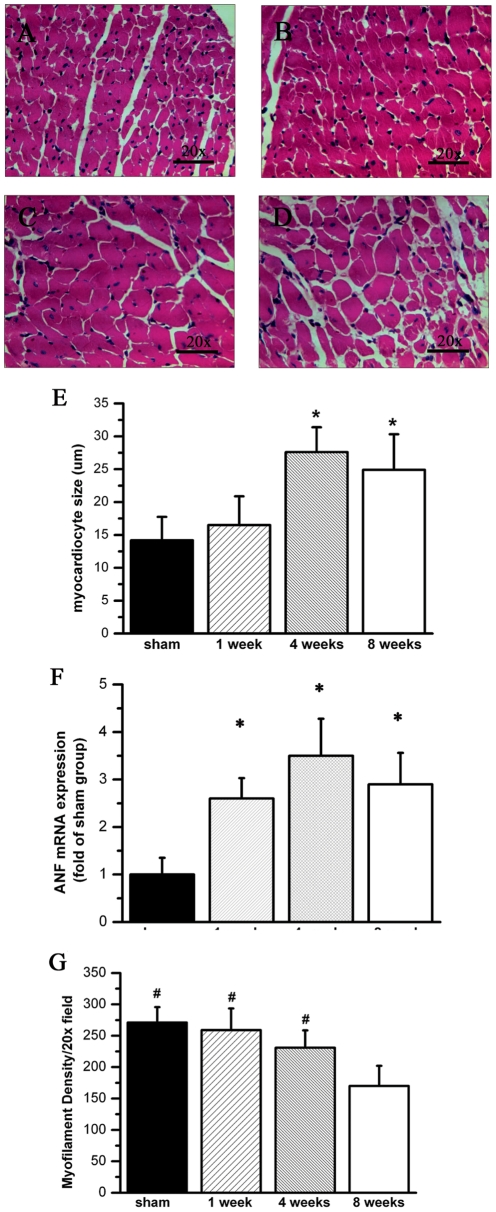
Myocytes in remote myocardium are hypertrophy and loss after infarction. A to D, Representative tissue slide from remote myocardium with HE in sham (A), one week (B), four weeks (C) and eight weeks group (D); E, Accumulated results show myocytes size significantly increases four wks and eight wks post MI; F, Accumulated results show ANF, which is the hypertrophic factor, is up-regulated after MI. G, Accumulated results show myofilament density is significantly decreased eight weeks post MI (n = 4). # P<0.05 compared by group of 8 wks post MI. * P<0.05 compared by sham group.

Because microvessel was crucial for myocytes alive, whether decreased micovessel resulted in hypertrophical myocytes loss needed to be elucidated. Thus we observed pathological alternation of myocytes in non-ischemic myocardium by hematoxylin-eosin staining. A point-to-point perpendicular line was drawn across the cross-sectional area of the myocytes at the level of the nucleus as previous described [15]. Significantly hypertrophy and loss of myocytes in the remote zone (LV posterior wall) was observed 8 weeks post MI compared with sham. Myocyte size was significantly increased 4 weeks and 8 weeks post MI (Fig. 4E). We also detected hypertrophy gene atrial natriuretic factor (ANF) expression by realtime PCR. We observed ANF mRNA expression was significantly increased after MI (Fig. 4F). While myofilament density in remote zone, which was defined as the total number of myocardiocytes per field, was normal 4 weeks post MI, and markedly decreased 8 weeks post MI (Fig. 4G). All these results indicated myocyte became hypertrophy in remote myocardium after MI. When neovasculerization dysfunction occurred 8 weeks after MI, myocytes was dead and cell density was decreased.

### ILK overexpression rescues heart failure by angiogenesis

To further verify that down-regulation of ILK accelerated cardiac remodeling and heart dysfunction via affecting tissue angiogenesis; we observed the therapeutic effect of sustained ILK expression in heart failure. To the aim, Ad-ILK was injected into remote myocardium to induce ILK expression continually 4 weeks after MI. Ad-null was used to inject as control. Two weeks after operation, we observed better heart function in ILK expressed rats after MI (Table 2). Ad-ILK treated rats have significantly higher FS level and smaller LVESD compared with those in Ad-null treated rats. The marked difference of FS and LVESD between Ad-ILK treated and control group indicated that ILK sustained rather than temporarily expression could slow down heart function deterioration after MI. Other parameters such as LVEDD and LVPWd revealed no difference between these two groups.

**Table 2:** Heart function after MI was perserved in Ad-ILK treated hearts (n = 6).

	4 week		6 week	
	control	ILK	control	ILK
**LVEDD(mm)**	7.25±0.11	7.176±0.12	8.09±0.08	8.06±0.11
**LVESD(mm)**	5.10±0.05	5.04±0.06	6.05±0.07	5.68±0.07*
**IVSd(mm)**	1.18±0.01	1.18±0.01	1.15±0.01	1.15±0.01
**LVPWd(mm)**	1.20±0.01	1.20±0.01	1.18±0.01	1.27±0.01
**FS(%)**	29.67±0.85	29.74±1	25.24±0.24	29.51±1.33*

*P<0.05 compared by sham group at each time point.

**Figure 5:**
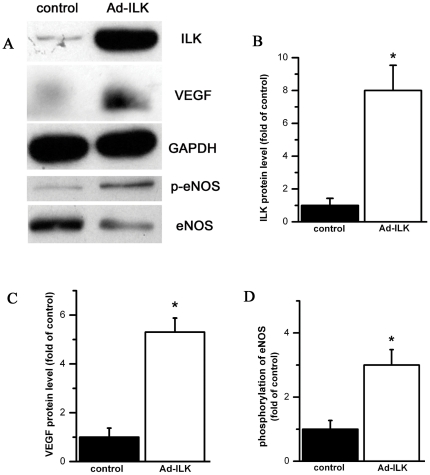
when ILK expression is increased by gene tranfected, angiogenesis signal pathway is activated in myocardium. A, Representative western blot results of ILK, VEGF and p-eNOS in the myocardium from Ad-ILK treated and control groups; B, Accumulated results show ILK expression is increased in Ad-ILK treated myocardium; C and D, VEGF and p-eNOS level is also increased in Ad-ILK treated myocardium (n = 6). P<0.05 compared by control group.

After ECG, myocardium was harvest for western blot analysis. We observed marked increased ILK expression in Ad-ILK treated myocardium (Fig. 5B). In ILK sustained expressed myocardium, VEGF expression (Fig. 5C) and phosphorylation of eNOS (Fig. 5D) were also significantly increased, indicating prolonged angiogenesis pathway activation in ILK sustained expressed myocardium. All of these results demonstrated once ILK expression was recovered, the angiogenesis related molecular was up-regulated again and heart function was preserved.

## Discussion

In present study, we observed that ILK expression was temporarily increased in non-ischemic myocardium by infarction, and decreased from 4 to 8 weeks post MI. During this period, the heart function of experimental MI rats was shift from compensatory to decompensatory. Further analysis revealed that down-regulation of ILK caused VEGF dependent angiogenesis dysfunction and myocytes loss in remote myocardium. Sustained expression of ILK by Ad-virus delivery could effectively promote angiogenesis and preserve heart function.

After myocardial infarction, non-ischemic myocyte hypertrophy is an adaptive response to increased external load, but sustained overload eventually results in progression from adaptive hypertrophy to failure. In previous works, heart failure of rat is established 6 to 9 weeks after myocardium infarction [16], [17], [18]. In present study, though LV dilation is sustained after MI, dp/dt_max_ and LVEDP are preserved until 4 weeks post-MI, and significantly deteriorated 8 weeks post-MI. These results indicate heart function is compensatory until 4 weeks in our animal model, but goes to failure 8 weeks post MI.

One important component to preserve cardiac function after MI is neovasculerization in remote myocardium [19], [20]. Insufficient microvessel adaptation in related to the degree of myocyte hypertrophy is an important reason for cell death, leading to progressive cardiac dysfunction in HF. In previous study, an adenoviral vector encoding the ectodomain of fetal liver kinase 1(FLK1), which inhibits VEGF related angiogenesis, is used in transverse aortic constriction (TAC) mice. Impaired VEGF pathway causes that angiogenesis does not keep pace with myocyte hypertrophy, contractile dysfunction and heart failure is accelerated [4]. In adiponectin knockout mice, which angiogenesis is interrupted, similar result is observed [21]. Other reports also reveal that microvessel density eventually decreases when heart failure occurs [22], [23]. In our study, p-eNOS/eNOS are increased until 4 weeks, and significantly decreased 8 weeks post-MI. In consistent with downregulation of pro-angiogenic signaling, histology study also shows microvessel density and myocyte number are reduced simultaneously 8 weeks post MI. Therefore we suggest that in the process of cardiac remodeling, impaired ILK related angiogenesis is the key feature when heart failure occurs.

Under LV strength stimulation, ILK is thought to promote tissue neovasulerization by VEGF. In present study, VEGF is observed to increase temporarily and return to normal 8 weeks post MI. Similar VEGF expression pattern is reported by previous work. In remote zone of experimental MI rat, VEGF level is increased in a time dependent pattern until about 40 days post operation [22]. These results demonstrate VEGF expression alternated in the process of cardiac remodeling. However unlike infarction zone, there is no ischemia in the remote myocardium. Therefore the VEGF expression would not depend on hypoxia related signal pathway, other mechanism would exist. The VEGF expression in our study is in accompany with ILK. ILK is an important protein to transduce mechanical stress to molecular signaling events in cell. It could interact with PI3K to phosphorylate Akt [24]. In tumor tissue, ILK is observed to induce VEGF expression by Akt-mTOR dependent pathway [10]. In our previous work, overexpression of ILK is observed to induce angiogenesis in heart by Akt activation [11]. Here, we observe temporally upregulation of ILK and VEGF simultaneously after infarction, indicating that ILK initiated VEGF expression in non-ischemic myocardium. When the external preload prolonged, ILK expression eventually returned to normal, and caused responsible VEGF expression attenuation. To further verify this mechanism, we induce ILK sustained expression 4 weeks after MI. When the pathological down-reguation of ILK is inverted, the VEGF in myocardium is never decreased and heart function is significantly improved.

There are many limitations in our study. First, in our animal model, heart function is compensatory until 4 weeks post MI by non-ischemic myocardium hypertrophy. But no increased ventricular wall thickness at 4 weeks post MI was observed. The reason should be insufficient resolving power of rat echocardiogram and sample number is relative small. Second, we further observe that ILK downstream kinase Akt is phosphrylated in non-ischemic myocardium after MI. But p-Akt level is not changed when ILK level is decreased. Akt could be phosphrylated by several kinase, such as ILK, PKC, ATM kinase as well as mTORC2 in a PI3K dependent manner [25]. Therefore, consistent with previous report [26], Akt could be still activated by other signaling pathway in heart failure, though ILK expression and activity is already decreased. On the other hand, ILK induced VEGF expression would not depend on Akt pathway in myocyte, further work needs to elucidate this issue.

In present study, we demonstrated that ILK could induce VEGF expression and a beneficial angiogenesis to maintain cardiac hypertrophy. Once ILK expression was decreased, VEGF dependent angiogenesis was attenuated, leading to myocyte death and heart failure. Furthmore sustained ILK expression could maintain angiogenesis in hypertropical myocardium, and preserve heart function after MI. Therefore ILK expression alternation was a critical pathophysiological feature that contributes to the transition from adaptive cardiac hypertrophy to cardiac dysfunction. Understanding the ILK related mechanism of cardiac maladaptation may lead to a new strategy for treatment of heart failure after infarction.
